# Scalable Moiré Lattice with Oriented TMD Monolayers

**DOI:** 10.1186/s11671-022-03670-y

**Published:** 2022-03-14

**Authors:** Meng-Hsi Chuang, Chun-An Chen, Po-Yen Liu, Xin-Quan Zhang, Nai-Yu Yeh, Hao-Jen Shih, Yi-Hsien Lee

**Affiliations:** 1 Department of Materials Science and Engineering, National Tsing Hua University, Hsinchu, 30013 Taiwan; 2 Frontier Research Center on Fundamental and Applied Sciences of Matters, National Tsing Hua University, Hsinchu, 30013 Taiwan

**Keywords:** Transition metal dichalcogenide, Scalable, Epitaxial monolayer, Moiré superlattice, Interlayer exciton

## Abstract

Moiré lattice in artificially stacked monolayers of two-dimensional (2D) materials effectively modulates the electronic structures of materials, which is widely highlighted. Formation of the electronic Moiré superlattice promises the prospect of uniformity among different moiré cells across the lattice, enabling a new platform for novel properties, such as unconventional superconductivity, and scalable quantum emitters. Recently, epitaxial growth of the monolayer transition metal dichalcogenide (TMD) is achieved on the sapphire substrate by chemical vapor deposition (CVD) to realize scalable growth of highly-oriented monolayers. However, fabrication of the scalable Moiré lattice remains challenging due to the lack of essential manipulation of the well-aligned monolayers for clean interface quality and precise twisting angle control. Here, scalable and highly-oriented monolayers of TMD are realized on the sapphire substrates by using the customized CVD process. Controlled growth of the epitaxial monolayers is achieved by promoting the rotation of the nuclei-like domains in the initial growth stage, enabling aligned domains for further grain growth in the steady-state stage. A full coverage and distribution of the highly-oriented domains are verified by second-harmonic generation (SHG) microscopy. By developing the method for clean monolayer manipulation, hetero-stacked bilayer (epi-WS_2_/epi-MoS_2_) is fabricated with the specific angular alignment of the two major oriented monolayers at the edge direction of 0°/ ± 60°. On account of the optimization for scalable Moiré lattice with a high-quality interface, the observation of interlayer exciton at low temperature illustrates the feasibility of scalable Moiré superlattice based on the oriented monolayers.

**Supplementary Information:**

The online version contains supplementary material available at 10.1186/s11671-022-03670-y.

## Introduction

Moiré lattice formed in the stacked monolayers of two-dimensional (2D) materials effectively modulates the electronic structures of materials, which raise considerable attentions in condensed matter physics and material sciences. Coupling among various electronic structures of the artificially stacked 2D lattices enables unique properties, such as non-conventional superconductivity [1, 2] quantized states of the interlayer exciton [3–6], and quantum emitting arrays [7] at a specific stacking and small twisting angle. With a precise twisting angle control of the hetero-stacked bilayer, the formation of Moiré superlattices promises the prospect of uniformity among different electronic moiré cells across the entire crystalline lattice. Currently, the Moiré 2D lattice has been mainly achieved by stacked bilayer of the exfoliated monolayers with a limited crystal size [8–11] or the synthesized monolayers with random orientation [12, 13], resulting in a limited size of the Moiré lattice. Fabrication of scalable Moiré lattice remains challenging due to the lack of essential manipulation of the highly-oriented monolayers. It is required to develop a clean and robust method to hetero-integrate the scalable monolayers with twisting angle control for scaling the Moiré 2D lattice [14, 15].

Recently, epitaxial growth of the monolayer TMD was achieved on the sapphire substrate by chemical vapor deposition (CVD) to realize scalable synthesis of the highly-oriented monolayers [16–21]. Hetero-epitaxial growth of the highly-oriented monolayer has been highlighted and widely studied for understanding of the fundamental mechanism to control the material orientation well. The well-aligned domains of the 2D materials were achievable by controlling rotation of the as-grown domains in the initial growth [22, 23] and step edges of the single crystalline substrate [24–28]. Epitaxial growth of the monolayer molybdenum disulfide (MoS_2_) was optimized with the position for high S/Mo precursor concentration ratio by two-step growth at specific temperatures [19]. An additional heating stage at a lower temperature for the nucleation process promoted rotations of the grown nuclei-like domains to align the underneath single crystalline substrate for a highly-oriented film. However, more experimental studies on the growth are required for new materials and a better understanding of fundamental mechanisms. Moreover, hetero-integrating the highly-oriented monolayers for various hetero-stacked bilayers provides an opportunity to scale Moiré lattice, which is essential for exploring novel properties with the Moiré superlattice.

In this study, epitaxial growth of the highly-oriented TMDs (WS_2_ and MoS_2_) is achieved with a customized CVD process. A controlled growth is observed with separated growth stages at different working temperatures. A full coverage and distribution of the highly oriented domains are verified by second-harmonic generation (SHG) microscopy. A large-area Moiré lattice is fabricated by stacking the highly-oriented monolayer TMDs (epi-WS_2_/ epi-MoS_2_) with a small twisting angle and a clean manipulation method. Photoluminescence (PL) measurement of the scalable Moiré is carried out at a low temperature of 77 K to verify the interlayer exciton and interface quality of the hetero-stacked bilayer.

## Methods/Experimental

### The Growth of Epitaxial Monolayer Tungsten Disulfide (WS_2_)

Epitaxial monolayer WS_2_ was synthesized on the sapphire substrate by the ambient-pressure chemical vapor deposition (APCVD). Solid precursors, including WO_3_ (600 mg, Alfa Aesar, 99.8% CAS# 1314-35-8) and Sulfur (10 mg, Sigma-Aldrich 99.5% CAS#7704-34-9) powders, were used for the CVD synthesis. The as-grown sapphire substrates were placed face-down on the WO_3_ crucible in the middle of furnace and S crucible was located on the upstream within the heating belt. The furnace was heated to the growth temperature of 950 °C. A gas flow of the mixed H_2_/Ar was 2/20 sccm in the initial growth stage and then 5/120 sccm in the steady state stage. After keeping for 5 min, the furnace was shut down and natural cooling.

### The Growth of Epitaxial Monolayer Molybdenum Disulfide (MoS_2_)

Epitaxial monolayer MoS_2_ was synthesized on the sapphire substrate by the low-pressure CVD (LPCVD). The growth condition was under ~ 5 torr with a temperature range from 750 to 900 °C. Solid precursors, including MoO_3_ (50 mg, Alfa Aesar, 99.5% CAS#1313-27-5) and Sulfur (500 mg, Sigma-Aldrich 99.5% CAS#7704-34-9) powders, were used for the CVD synthesis. The as-grown sapphire substrates were placed face-up in the middle of furnace at the downstream of the sequential S and Mo crucibles. Ar gas flow ~ 200 sccm was blown for an inert atmosphere. The furnace was heated to achieve a temperature of 800 °C, for a relatively low nucleation and then further heated to achieve the growth temperature of 950 °C. Relative to multi-stage heating process for Mo powders, the sulfur was directly heated to 250 °C in the beginning of the experiment to maintain the sufficiency of sulfur precursors concentration. After keeping for 5 min, the furnace was shut down and naturally cooled.

### PDMS Stamping for Clean Manipulation

The PDMS stamps were prepared using the mixed prepolymer and cross-linker at a weight ratio of 10:1 with a curing time of 48 h at room temperature under vacuum situation. During the transfer, the PDMS stamp was first placed on an as-grown monolayer. After submerged into deionized water, the PDMS was carefully lifted and placed TMDs side up on a quartz slide. The quartz slide was then being fixed under the optical microscope with a XYZ rotatable stage beneath. The target sample was brought in contact with the PDMS stamp by the stage at the desirable position and twisting angular degree, resulting in a stacking hetero-stacked bilayer. Finally, the stacked sample was heated to 70 °C, causing the PDMS to lose its stickiness before removed.

### Material Analysis

Optical microscopy (OM) and Atomic force microscopy (AFM) are used to characterize the surface morphology and microscale structure for epi-TMD films. The highly-oriented domains and their morphologies are observed by the optical microscopy (Olympus-BX51). We could distinguish the monolayer TMD by a clear contract and verify the various of thickness with the color of different shades for the initial judgment. Scanning Probe Microscopy is performed by Bruker Dimension® Icon with basic tapping mode height profile measurement by TESPA tips.

### SHG Nonlinear Microscopy

A customized nonlinear microscope, based on Leica SP5 confocal microscope is operated in non-descan mode and equipped with motorized tuning capabilities in in-plane directions (*x*, *y*) and azimuthal rotation (*φ*). The wavelength of fundamental wave (FW) laser is set to 810 nm for the whole SHG measurement and focused on the sample with a 10 × objective lens. Through this setup, SHG mapping over large-area region could be efficiently obtained. While rotating the sample, which is fixed on the stage, angle (${\mathrm{\varphi }}_{\mathrm{FW}-D}$) between the polarization of incident FW laser and the armchair direction of the monolayer TMD domain can be adjusted.

### Raman and PL Microscopy

Raman and micro-PL spectroscopy are utilized with a confocal microscope system, equipped with a 532 nm continuous wave (CW) pumping laser at room temperature. (Micro Raman/PL/TR-PL Spectrometer, Ramaker, Protrustech). 50 × Long working distance objective lens are used with a spot size of the laser around 1–2 $\mathrm{\mu m}$ for full-range temperature experiment from 77 K to room temperature. Temperature Control Systems for Microscopy and Spectroscopy are carried out to measure the optical properties under low temperature (THMS350V, Linkam Scientific). Typical gratings are used with 300 g/mm for PL (low resolution) to get broadband spectrum and 1800 g/mm for Raman (high resolution) signals to get the detail information of material.

## Results and Discussion

**Fig. 1:**
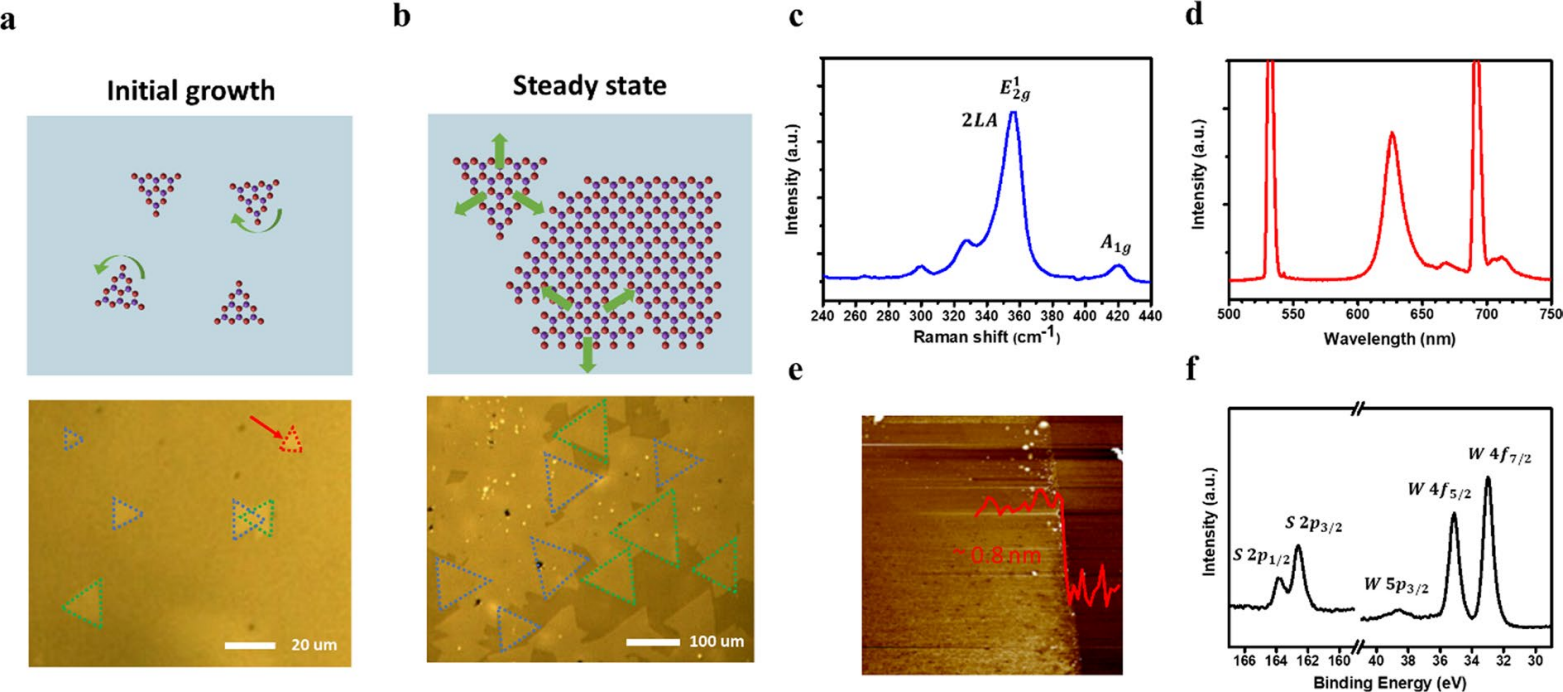
The scalable epitaxial growth of highly-oriented monolayer TMDs by customized CVD process. Schematics illustration of growth mechanism in the **a** initial growing stage and **b** steady state stage with related OM images. **c** Raman, **d** PL, **e** height profile and **f** XPS analysis of epitaxial monolayer WS_2_

Van der Waals (vdW) epitaxy was achieved for the highly-oriented TMD monolayer by controlling surface structures of the single crystalline sapphire substrate with a reduced formation energy under certain surface termination [29–31] and small lattice mismatch [17, 18]. With our customized CVD process, the monolayer TMDs (MoS_2_ and WS_2_) are epitaxially grown on the sapphire with two major orientations (0°/ ± 60° to the edge). The growth of highly-oriented TMDs is achieved and optimized by controlling growth conditions in the two representative growth periods, as shown in Fig. 1a, b, respectively. Two stages including nuclei-like domain rotating in the initial growing stage and grain size extending in the steady state stage dominate the synthesized process and the schematics of the growth mechanism are demonstrated. In the initial growth stage, some misoriented nuclei-like domains are observed and rotatable with reaction of small amounts of precursors at a relatively lower temperature. In Fig. 1a, the rotation only appears at a low temperature of 800–850 °C with a smaller domain size, leading to a pair of orientations for the lowest energy states. With the increase in working temperature, the growth behavior prefers lateral growth than formation of more nuclei-like domains by suitable control of reacting conditions. In the steady state growth stage, lateral growth of the aligned domains appears and forms a full coverage of highly-oriented monolayer TMD at a high temperature of 950 °C, as shown in Fig. 1b. With the optimized conditions, the aligned domains become non-rotatable in the stage of steady-state growth, enabling the vdW epitaxy for the synthesized monolayer TMD with aligned domains at a pair of the preferred orientations at 0° and ± 60°.

To obtain more highly-oriented monolayer TMDs for scalable Moiré lattice of the stacked bilayer, monolayer of highly-oriented MoS_2_ was epitaxially grown on the sapphire substrate, which was consistent to the reported work [19]. To enable vdW epitaxy of the MoS_2_ for highly-oriented monolayer, a setup of low-pressure CVD is effective for optimizing the growth in the sufficiency of sulfur vapor concentration (detailed information is described in the “method/experimental” section). Additional file 1: Figure S1 demonstrates the statistical analysis for the aligned domains (at 0° and ± 60°) of the large-area monolayer MoS_2_ epitaxially grown on sapphire substrates. Transitions from randomly-oriented to well-aligned domains were realized by promoting the rotation for aligned domains in the initial growth and sequentially growing in the second growth stage at a higher growth temperature. In contrast, epi-WS_2_ film was rarely synthesized before due to the low WO_3_ vaporizing pressure and bad reactivity. Large-area monolayer WS_2_ film was epitaxially grown on sapphire substrates by using ambient-pressure CVD with a heating belt system, as shown the schematic illustration in Additional file 1: Figure S2a). Reduced concentration of the precursors and a high ratio (S/W) of the chalcogen to transition metals, achieved with an increased Ar gas flow in the steady state stage and a controlled sulfur vapor in the initial growth stage, significantly promoted the vdW growth for the highly-oriented monolayer TMD. Additional file 1: Figure S2b–e presents the influence of H_2_ flow on the lateral growth of highly-oriented monolayer WS_2_ to grow the epitaxial film. Similar to the growth of the MoS_2_, synthesis of the highly-oriented WS_2_ was optimized by controlling growth parameters for enhanced nucleation and growth of the monolayer with a larger domain size and higher quality.

In Fig. 1b, optical microscope (OM) image of the large-area and highly-oriented monolayer WS_2_ indicated that the aligned domains were over 90% coverage with an average size of 100–200 $\mathrm{\mu m}$. Figure 1c shows the characteristic Raman spectrum where the vibration mode of 2LA(M), E_2g_, and A_1g_ peak are located at 351 cm^−1^, 355 cm^−1^, and 417 cm^−1^, respectively, suggesting the high-quality monolayer crystal structure. Photoluminescence (PL) spectrum with the peak position of 626 nm exhibited the optical property of the direct band gap structure, as shown in Fig. 1d. In addition, the thickness of monolayer epi-WS_2_ was verified ~ 0.8 nm with the atomic force microscopy (AFM) as presented in Fig. 1e. Figure 1f shows the binding energy of S 2*p* and W 4*f* core level for the X-ray photoelectron spectroscopy (XPS) analysis and no obviously additional peaks exhibit that the material was relatively uniform without damage and oxidation.

**Fig. 2:**
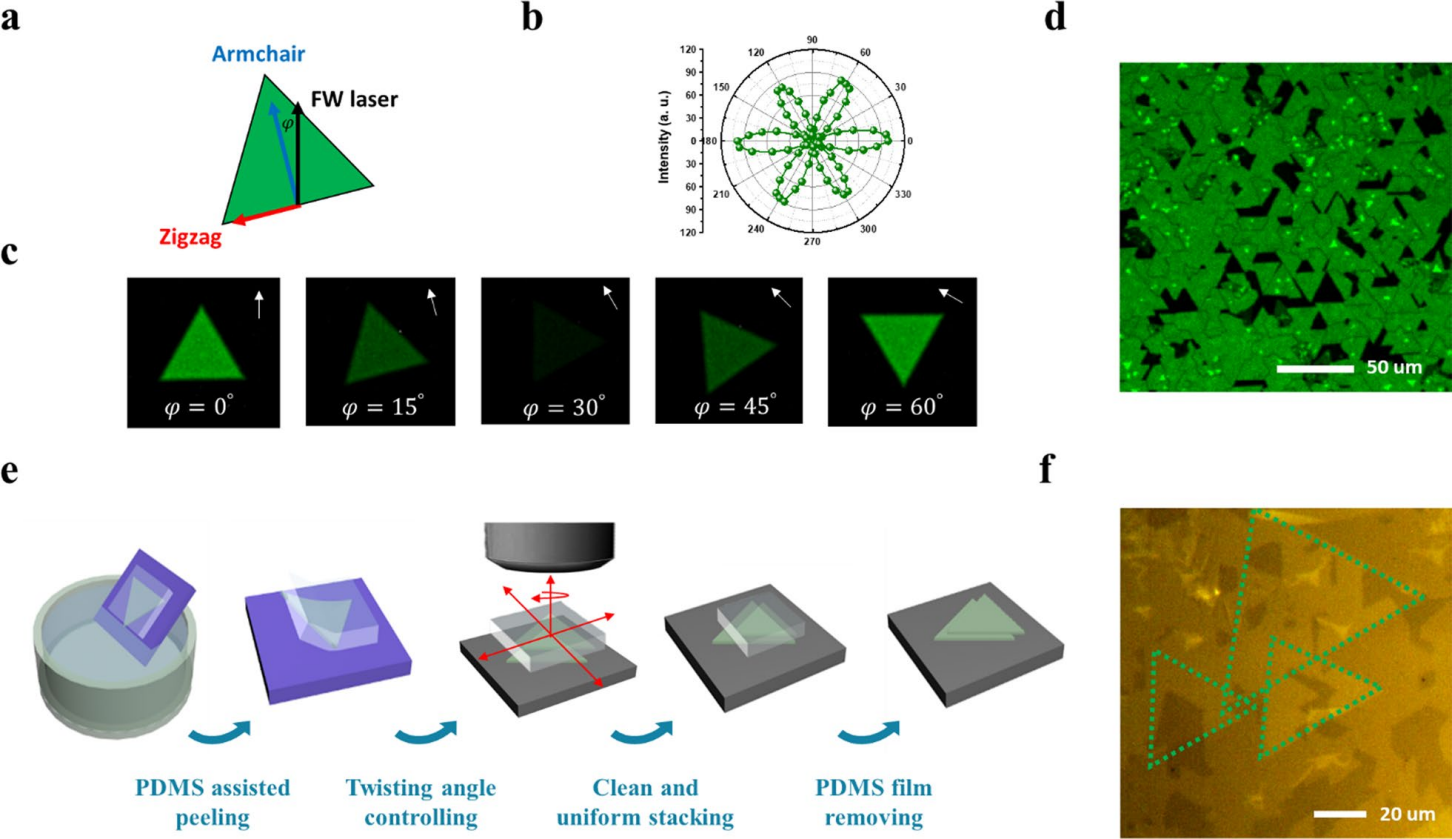
Integrating the oriented TMD for scalable Moiré lattice with twisting control. **a** Schematic illustration of TMD with zigzag and armchair direction, while *φ* is defined as the angle between the polarization of incident FW laser and the armchair direction of the TMD. **b** Polar plot of polarization resolved SHG intensity. **c,** The SHG mapping images for monolayer MoS_2_ grain with misaligned angles ($\varphi_{{{\text{FW}} - D}}$) between 0° and 60°. **d** The SHG intensity mapping of epi-TMD film. **e** Schematics illustration of clean water transfer technique with PDMS stamping. **f** OM images of the stacked hetero-bilayer

Distribution on the orientation of the single crystalline domains in the TMD materials determines the symmetry of artificially stacked bilayer, which is significant to entire band structures and nonlinear optical properties [32–34]. Second harmonic generation (SHG) microscopy is adopted to further verify the spatial variations of the orientation in the scalable and highly-oriented monolayer TMDs. The 2H phase of the monolayer TMD (MoS_2_ and WS_2_) exhibits D_3h_^1^ symmetry group with a broken inversion symmetry, which enables a high second-order nonlinear optical susceptibility [35, 36]. While rotating the sample, angular degree (${\mathrm{\varphi }}_{\mathrm{FW}-D}$) between the polarization of incident FW laser (black arrow) and the armchair direction of the TMD domain (blue arrow) can be adjusted as shown in Fig. 2a. The emitting SHG intensity shows an angular dependence of ${\mathrm{cos}}^{2}(3 {\mathrm{\varphi }}_{\mathrm{FW}-D})$ with respect to the FW laser polarization (Fig. 2b). The brightness evolution, shown in Fig. 2c, is in good agreement with the measured polarization-resolved SHG emission. With the mapping of the SHG intensity, the single crystalline domain exhibits the same intensity at the edge orientation of 0° and ± 60°, while grains with other orientation angles can be easily distinguished because of the darker emissions. Such polarization-sensitive optical nonlinearity is powerful on the determination of the twisting angle. Figure 2d and Additional file 1: Figure S3 show the SHG intensity map with scaled color contour between an epitaxial and a non-epitaxial film. Statistical analysis from intensity map exhibited that the preferred orientation for the as-grown epi-MoS_2_ increased from 80% with half coverage up to over 95% with full coverage. The result motivates further efforts to focus on the development of Moiré hetero-stacked bilayer with coherently stacking.

The large-area Moiré superlattice was stacked by transferring the scalable highly-oriented WS_2_ on the as-grown scalable highly-oriented MoS_2_ with the polydimethylsiloxane (PDMS) assisted water transfer technique [37–40] for interfacial cleanness and precise twisting angle control. A transparent, flat and flexible PDMS film was adopted as a supporting layer and the stacking sample (PDMS/epi-TMD film) was gently lifted-off from the as-grown substrates after immersing into the deionized water. The PDMS/material film was then fixed on the quartz slide under the XYZ rotatable stages system, and precisely stamped on the highly-oriented heterostructure at the target substrate with a desirable position and twisting angle, resulting in an angle-controlled hetero-stacked bilayer. Finally, the stacked sample was heated to 70 °C and the PDMS thin film without stickiness was peeled off with clean surface. The schematic transfer process is illustrated in Fig. 2e and more detail is shown in the “method/experimental” section. Since the bottom layer of highly-oriented monolayer TMD stays atomically flat on the as-grown substrate and the sequential transferred monolayer epi-TMD is stacked on top of that by clean water transferring, the large-area hetero-stacked bilayer would be achieved with a high interfacial quality and ultra-clean surface. Figure 2f shows the OM image of a hetero-stacked bilayer for epi-WS_2_/epi-MoS_2_. The optical image demonstrated the uniformity and cleanness throughout the stacked epitaxial monolayers. Furthermore, the precision of the bilayer twist angle could be assured from the sharp edges of the two TMD materials.

**Fig. 3:**
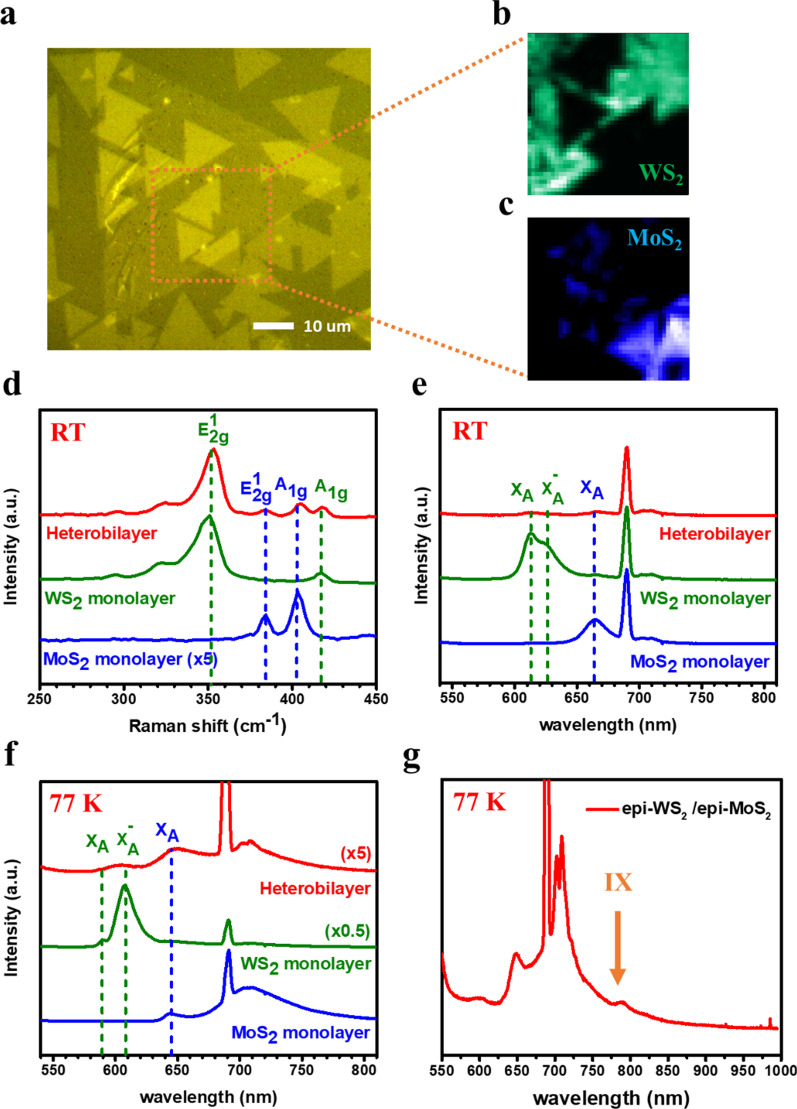
Strong interlayer coupling at the stacked bilayer of the oriented monolayers. **a** OM image of the stacked bilayer of the epi-WS_2_ and the epi-MoS_2_. PL mapping of **b** epitaxial monolayer WS_2_ and **c** epitaxial monolayer MoS_2_. **d** Raman and **e** PL spectra at room temperature. **f** PL spectra and **g** interlayer exciton emission at low temperature of 77 K

To study optical properties of the hetero-stacked bilayer, Raman and micro-PL spectroscopy were obtained with a confocal microscope system. Figure 3d shows the Raman spectra of the highly-oriented monolayer TMDs (MoS_2_ and WS_2_) which are compared with that of the stacked WS_2_/MoS_2_ heterostructures (Fig. 3a) on the sapphire substrate. Two Raman vibration peaks were revealed at 383 and 404 cm^−1^, corresponding to the in-plane E_2g_ and out of plane A_1g_ vibrational modes of MoS_2_, while the main feature peaks located at 351, 355, and 417 cm^−1^, corresponding to second-order mode of longitudinal acoustic phonon 2LA(M), in-plane E_2g_, and out-of-plane A_1g_ of WS_2_, respectively. The Raman spectra measured in the heterostructure bilayer contained both the characteristic peaks of MoS_2_ and WS_2_. Furthermore, we observed that the peak position of the Raman A_1g_ modes stiffened about 2–3 cm^−1^ in the stacked samples attributed to strong interlayer coupling, which was consistent with previous papers [39, 41, 42]. Figure 3e similarly collects the PL signals from epi-films of MoS_2_, WS_2_, and the hetero-bilayer area at room temperature, respectively. Strong photoemission displayed at 667 nm (~ 1.83 eV), which corresponds to the A excitonic transitions for MoS_2_, while two peaks located at 615 nm and 626 nm, which corresponds to the exciton and trion emission for WS_2_. The PL spectra measured at room temperature in the heterojunction region exhibited the obvious PL quenching phenomenon compared to the dual monolayer region, also indicating the cleanness of WS_2_ /MoS_2_ interface with strong interlayer coupling [41, 43, 44].

The low-temperature PL characterization at 77 K was performed to further identify the interface quality and performances of the scalable Moiré lattice. Both oriented monolayers of the WS_2_ and the MoS_2_ exhibited an obvious blue-shift of PL peaks, as shown in Fig. 3f. The A-exciton emission position of the MoS_2_ shifted from 667 to 646 nm, which can be attributed to the change in lattice size with the decrease in temperature. The spectral weight of WS_2_ shifted from exciton (590 nm) to trion (609 nm) indicating that the thermal energy at higher temperature is large enough to lead to a partial dissociation of the trions [45]. In the spectrum of the MoS_2_, a broaden peak at ~ 710 nm represents the defect peak of the epi-MoS_2_. The low temperature intensity mappings of MoS_2_ PL characteristic peak at 646 nm, and WS_2_ trion peak at 609 nm were shown, respectively, in Fig. 3b, c, corresponding to the field of view of the white rectangular region in Fig. 3a. The obvious quenching of both characteristic peaks was observed from the intensity map as the same interlayer effect at the room temperature. The magnified PL spectrum of the hetero-bilayer region, presented in Fig. 3f contained both the characteristic peaks, corresponding to direct excitonic transition energies in monolayer MoS_2_ and WS_2_ while the substrate peaks were located around 700 nm. Moreover, there was a weak peak at ∼ 790 nm (∼ 1.57 eV) which could be represented by the interlayer exciton emission (Fig. 3g). It is noteworthy that the evidence of interlayer exciton is visualized without the h-BN capping, suggesting a high quality of the hetero-interface. Inhomogeneity on interlayer interactions is due to variation of the hetero-interface at stacked monolayer. In future studies, scalable integration of the synthesized h-BN with the Moiré lattice would effectively improve the homogeneity of the interlayer interactions. These above observations confirm an ideal interface between the scalable and highly-oriented monolayer (WS_2_ and MoS_2_) for strong interlayer coupling of physical parameters, which move a significant step toward large-area Moiré heterostructure.

## Conclusions

Scalable highly-oriented monolayer TMDs (WS_2_ and MoS_2_) are hetero-epitaxially grown with customized CVD by controlling working temperature of growth stages. Controlled growth is achieved by promoting rotation of the nuclei-like domains with the growth parameters to well align the single crystalline substrates in the initial growth and growth of domains to the unidirectional film in the steady state. Coverage and distribution of the highly-oriented domains are verified by SHG microscopy. With the PDMS-assisted transfer method for a clean manipulation, large-area hetero-stacked bilayer of the two oriented monolayers (epi-WS_2_/epi-MoS_2_) is fabricated with a twisting angle control. The interlayer exciton observed at 77 K confirms the interface quality and uniformity of the Moiré lattice. Scalable Moiré lattices move a significant step toward artificial electronic structures of 2D lattices.

## Supplementary Information

**Additional file 1**. Scalable synthesis of epitaxial monolayer TMDs.

## Data Availability

All data generated during this study are included in this published article and its supplementary information files.

## Abbreviations

2D: Two-dimensional
TMD: Transition metal dichalcogenide
CVD: Chemical vapor deposition
OM: Optical microscope
AFM: Atomic force microscopy
PL: Photoluminescence
XPS: X-ray photoelectron spectroscopy
SHG: Second harmonic generation
