# Supplementary material for: Scalable Moiré Lattice with Oriented TMD Monolayers
Source: Nanoscale Res Lett. 2022 Mar 14;17:34. doi: 10.1186/s11671-022-03670-y (PMC8921411; doi:10.1186/s11671-022-03670-y)
Supplement: Supplementary file 1 — Additional file 1. Scalable synthesis of epitaxial monolayer TMDs. [file 11671_2022_3670_MOESM1_ESM.docx]

**Supporting Information**

**Scalable Moiré lattice with oriented TMD monolayers**

**Authors:** Meng-Hsi Chuang^1^, Chun-An Chen^1^, Po-Yen Liu^1^, Xin-Quan Zhang^1^, Nai-Yu Yeh^1^, Hao-Jen Shih^1^, Yi-Hsien Lee^1,2*^

**Affiliations:**

*^1^ Dept. Materials Science and Engineering, National Tsing Hua University, Hsinchu 30013, Taiwan.*

*^2^ Frontier Research Center on Fundamental and Applied Sciences of Matters, National Tsing Hua University, Hsinchu 30013, Taiwan.*

*Correspondence to: yhlee.mse@mx.nthu.edu.tw (YHL)

**
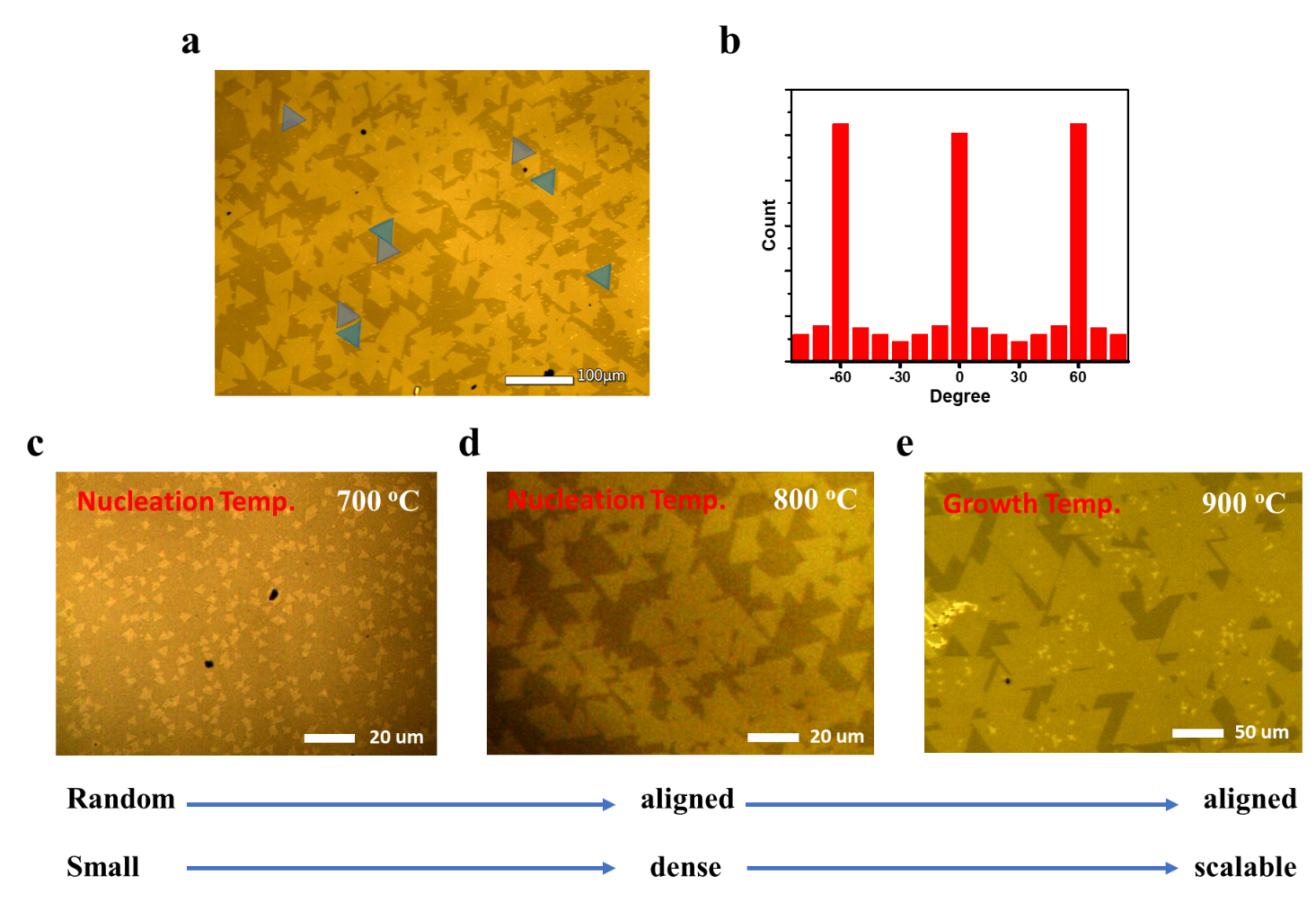
**

**Figure S1. Scalable synthesis of highly-oriented monolayer MoS_2_ on sapphire: a,** optical image of scalable epitaxial monolayer MoS_2_. **b,** the statistical analysis of the highly-oriented domains ($0^{\circ}/60^{\circ}$ to the edge). **c, d, e,** the temperature effect in the initial growth and the steady-state stage.

**
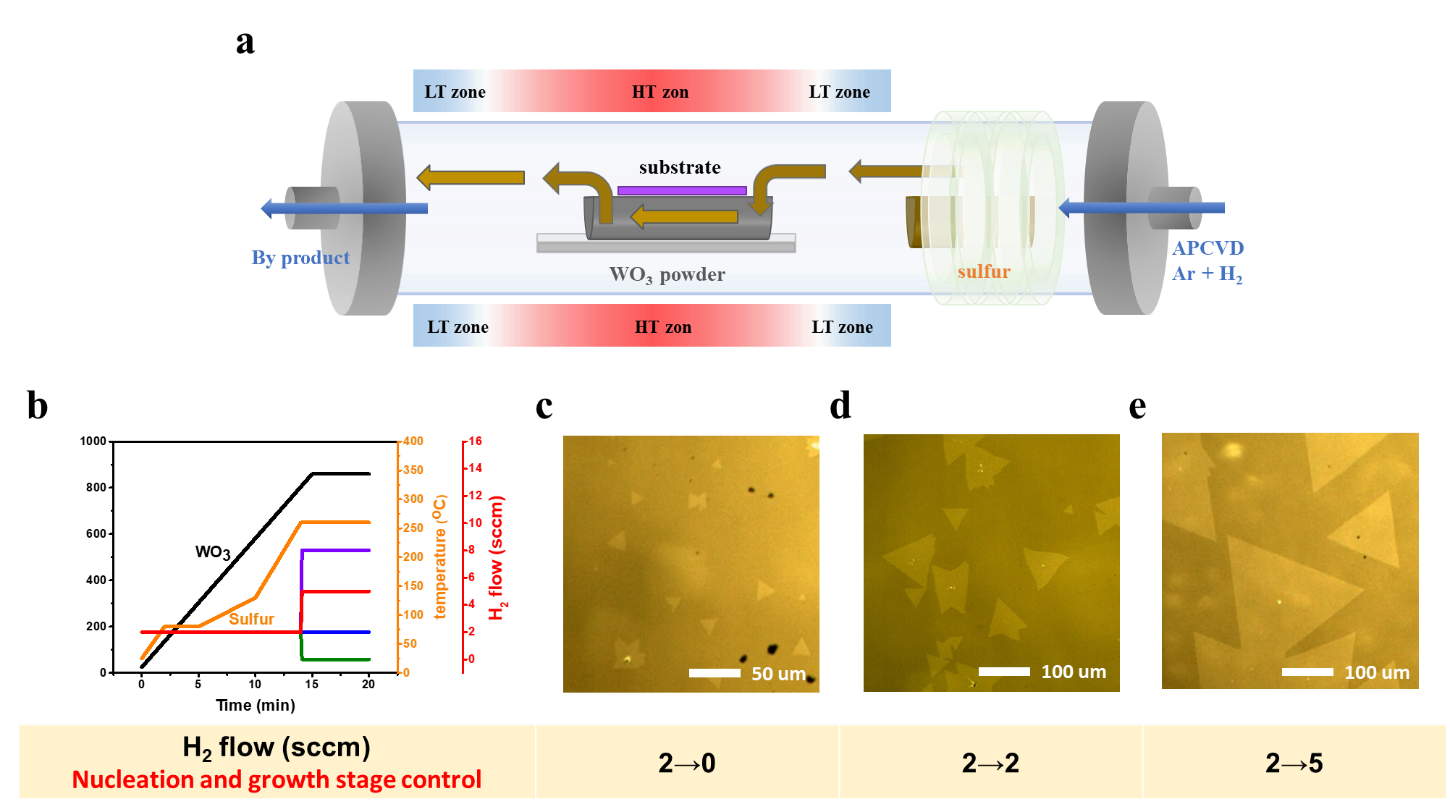
**

**Figure S2. Scalable synthesis of the highly-oriented monolayer WS_2_ on sapphire: a,** schematic illustration of the epitaxial growth of the highly oriented monolayer WS_2_ by APCVD. **b,** the heating process for the growth with the effects of the H_2_ flow and temperature. **c-e,** the influence of H_2_ flow in the steady-state growth stage (from 0 to 5 sccm).


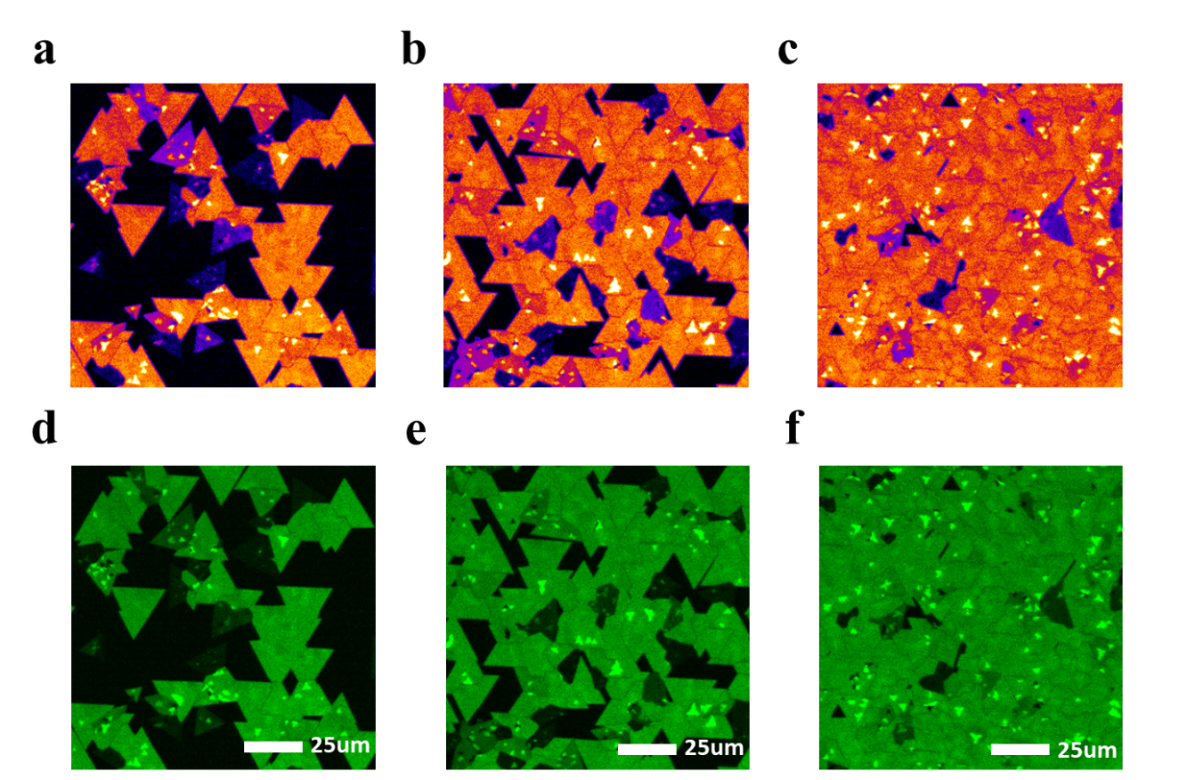


**Figure S3. Coverage-dependent SHG of the highly-oriented monolayer MoS_2_**

**a, d,** 50 % coverage. **b, e,** 81 % coverage. **c, f,** 98 % coverage.
